# Burden of Disease of Borderline Personality Disorder: A Comprehensive Evaluation of Quality of Life and Societal Cost of Illness

**DOI:** 10.1002/jclp.70000

**Published:** 2025-07-10

**Authors:** Carlijn J. M. Wibbelink, Arnoud Arntz, Jan H. Kamphuis, Iuno Z. Groot, Roland Sinnaeve, Silvia M. A. A. Evers

**Affiliations:** ^1^ Department of Clinical Psychology University of Amsterdam Amsterdam the Netherlands; ^2^ Academic Center for Trauma and Personality Amsterdam the Netherlands; ^3^ Department of Neurosciences, Mind Body Research KU Leuven Leuven Belgium; ^4^ Care and Public Health Research Institute (CAPHRI) Maastricht University Maastricht the Netherlands; ^5^ Department of Health Services Research Maastricht University Maastricht the Netherlands; ^6^ Centre for Economic Evaluation and Machine Learning, Trimbos Institute Netherlands Institute of Mental Health and Addiction Utrecht the Netherlands

**Keywords:** borderline personality disorder, burden of disease, cost‐of‐illness, quality of life, societal costs

## Abstract

Information on the burden of disease, including quality of life (QoL) and societal costs, of borderline personality disorder (BPD) is crucial, as healthcare policymakers consider the burden of disease when setting priorities for treatment reimbursement. We conducted a comprehensive evaluation of the burden of disease of BPD by estimating annual costs from a societal perspective using a bottom‐up approach and distinguishing between costs primarily related to psychological and somatic problems. QoL was determined using a generic QoL measure (EQ‐5D five‐level version [EQ‐5D‐5L]) as well as a measure specifically designed for individuals with psychological problems (Mental Health Quality of Life seven‐dimensional questionnaire [MHQoL‐7D]). Additionally, societal costs and QoL were compared with a comparison group. Data from 204 Dutch treatment‐seeking outpatients diagnosed with BPD and 86 individuals without severe psychological problems were analyzed. The results indicated a severely impaired QoL (EQ‐5D‐5L: 0.51, MHQoL‐7D: 0.24) combined with substantial societal costs (average total €35,038 per year) for BPD outpatients, markedly different from the comparison group. Societal costs of BPD were primarily attributable to psychological problems, with costs in other sectors as the main cost driver. The BPD group incurred higher costs for most patient and family cost items and cost items in other sectors, whereas differences in healthcare costs were limited to outpatient psychiatric treatment, consultations with general practitioners, emergency care, and social work. The high economic burden, along with the low QoL, suggests that increased treatment reimbursement for BPD would benefit both patients and society at large.

**Trial Registration:** The BOOTS study was registered in the Overview of Medical Research in the Netherlands (NL‐OMON21337), formerly known as the Netherlands Trial Register.

## 1 Introduction

Borderline personality disorder (BPD) is a severe disorder affecting between 1% and 3% of the general population and up to 22% in clinical populations (Ellison et al. 2018; Ten Have et al. 2016). Individuals diagnosed with BPD exhibit persistent patterns of emotional instability, identity disturbance, impulsive behavior, and interpersonal difficulties (American Psychiatric Association 2013). In addition, BPD is associated with a high risk of suicide (Paris 2019) and frequently co‐occurs with other mental disorders, including anxiety, mood and substance use disorders and other personality disorders (Bohus et al. 2021; IsHak et al. 2013; Leichsenring et al. 2023). Specialized psychotherapy is recommended as the primary treatment for BPD (Leichsenring et al. 2023). However, access to treatment is limited, leading to a significant portion of patients not receiving psychotherapy (Choi‐Kain et al. 2016; Iliakis et al. 2019; Hermens et al. 2011; Tusiani‐Eng and Yeomans 2018). Lack of access is mainly due to constraints in terms of resources, including time, qualified staff, and budget (Drummond et al. 2015; Hermens et al. 2011; Tusiani‐Eng and Yeomans 2018). Psychotherapy for BPD typically involves multiple sessions per week for 1–3 years and requires highly trained professionals (Hermens et al. 2011; Tusiani‐Eng and Yeomans 2018), making it an expensive treatment. Healthcare policymakers consider the burden of disease of patient populations when setting priorities for treatment reimbursement (Stolk et al. 2005). A high burden of disease, in terms of quality of life (QoL) and economic costs, can justify prioritizing the reimbursement of costly interventions (Norheim et al. 2014). Therefore, studying the burden of disease associated with BPD is of pivotal importance, and forms the focus of the current study.

BPD has a profound impact on several life domains. Most individuals diagnosed with BPD report severe functional impairments across interpersonal, occupational, academic, and financial domains (Ansell et al. 2007; Jørgensen et al. 2009; Skodol et al. 2002; Winograd et al. 2008), and these impairments are higher in BPD than in other personality disorders or major depressive disorder (Ansell et al. 2007; Skodol et al. 2002; Zanarini et al. 2010). Individuals with BPD are frequently unemployed and receive welfare or disability benefits, which poses a significant burden on society (van Asselt et al. 2007; Wagner et al. 2022). Moreover, BPD is associated with extensive use of psychiatric services, including frequent and prolonged outpatient and inpatient treatments (Bender et al. 2006; Salvador‐Carulla et al. 2014). In addition, use of psychotropic medication is common among BPD patients, with up to 96% of individuals with BPD receiving at least one psychotropic medication (Bridler et al. 2015). Somatic healthcare use is also elevated in BPD (Hastrup et al. 2019), likely due to the association between BPD and somatic conditions, including cardiovascular, respiratory, endocrine, metabolic, and sexually transmitted diseases (El‐Gabalawy et al. 2010; Schneider et al. 2019; Tate et al. 2022). Furthermore, BPD is related to an increased criminality rate (Sansone and Sansone 2009) and high out‐of‐pocket expenses, such as excessive shopping and substance use (van Asselt et al. 2007). The BPD diagnosis also places a significant burden on loved ones, as evidenced by their increased burden and impaired empowerment along with symptoms of depression and anxiety (Bailey and Grenyer 2013; Hastrup et al. 2019).

The chronic and complex nature of BPD, affecting multiple aspects of patients' lives, is also reflected in their QoL and societal costs. Research published to date presents a low QoL in individuals with BPD together with high economic costs (e.g., Bode et al. 2017; IsHak et al. 2013; van Asselt et al. 2007; Wagner et al. 2022). QoL among BPD patients is often expressed in utility scores generated by the EQ‐5D (Dolan 1997), which reflects a value the public would assign to a person's health state, ranging from 0 (a health state equivalent to death) to 1 (perfect health) (IsHak et al. 2013). Utility scores in BPD vary around 0.5, indicating a severe burden of disease (range: 0.47–0.52; Bales et al. 2012; Laurenssen et al. 2016; McMain et al. 2012; Sinnaeve et al. 2018; Soeteman et al. 2008; van Asselt et al. 2009). Moreover, this score is lower than observed in common mental disorders and comparable with the utility scores of severe somatic diseases (e.g., Parkinson's disease, stroke; Laurenssen et al. 2016). However, the use of the EQ‐5D to assess QoL in individuals with mental health problems has been criticized because of its limited ability to capture patient‐relevant outcomes (Brazier 2010) and for lacking sufficient congruence with the World Health Organization (WHO) dimensions of QoL (IsHak et al. 2013).

Studies examining the economic costs of BPD have consistently revealed high costs (Bateman and Fonagy 2003; Bode et al. 2017; Hastrup et al. 2019; Salvador‐Carulla et al. 2014; Sinnaeve et al. 2018; van Asselt et al. 2007; Wagner et al. 2014, 2022; Wunsch et al. 2014), considerably higher than observed in patients with other mental disorders (e.g., anxiety disorders, mood disorders) or physical disorders (e.g., Parkinson's disease, epilepsy) (Laurenssen et al. 2016). Nevertheless, considerable differences were found between studies, mainly related to methodological differences (Wagner et al. 2022). Moreover, previous research is limited by narrow perspectives, top‐down costing methods based on national statistics, and outdated results within the Dutch context. Additionally, most studies have only included costs related to the patient's psychological complaints, neglecting costs related to somatic problems. This narrow focus may well lead to an underestimation of BPD‐related costs (Wagner et al. 2022), as somatic problems in individuals with BPD could be a direct (stress, dysregulation of cytokines) or indirect (e.g., maladaptive lifestyle choices, iatrogenic factors) consequence of the disorder (Castle 2019). Finally, only one study compared societal costs of individuals with BPD to a comparison group (Hastrup et al. 2019). This is surprising, as comparing societal costs of BPD patients with that of individuals without severe psychological problems provides insight into the additional costs associated with BPD, beyond the average costs incurred by an individual. Moreover, it may also indicate the potential societal benefits of treating BPD.

Taken together, BPD not only severely impacts individual patients, as evidenced by low QoL, but also poses a high financial burden on society. However, previous research into the burden of disease of BPD has been subject to several limitations, such as the sole reliance on the EQ‐5D, inclusion of only costs related to psychological problems, and the absence of a comparison group. Given the importance of burden‐of‐disease studies in informing healthcare policy and prioritization (Jo 2014), a comprehensive evaluation of the burden of disease among individuals with BPD is warranted. Therefore, the aim of the present study was to gain insight into the burden of disease among Dutch treatment‐seeking outpatients with BPD by (i) estimating annual costs from a societal perspective through a bottom‐up (individual‐level) approach, and (ii) assessing the QoL using both the widely used EQ‐5D five‐level version (EQ‐5D‐5L; Herdman et al. 2011) and a novel measure specifically designed for people with mental health problems (Mental Health Quality of Life seven‐dimensional questionnaire [MHQoL‐7D]; van Krugten et al. 2022). The BPD outpatients were candidate participants in a multicenter randomized clinical trial (RCT) investigating the effectiveness of evidence‐based treatment for BPD (the Borderline Optimal Treatment Selection [BOOTS] study; Wibbelink et al. 2022). In addition, the results were compared with results of a comparison group consisting of individuals without severe psychological complaints.

## 2 Methods

We performed a bottom‐up, prevalence‐based, retrospective cost‐of‐illness study from a societal perspective, in which we also measured generic and disease‐specific QoL. The study adheres to the Consolidated Health Economic Evaluation Reporting Standards (CHEERS) 2022 statement (Husereau et al. 2022) and the consensus‐based checklist for cost‐of‐illness studies (Schnitzler et al. 2023).

### 2.1 Participants

The total sample included 204 participants with BPD seeking specialized outpatient treatment and 86 participants without severe psychological complaints. BPD participants were included if they met the following criteria: (1) BPD as the primary diagnosis, with a severity score above 20 on the Borderline Personality Disorder Severity Index version 5 (BPDSI‐5), (2) no diagnosis in the past year of a psychotic disorder or bipolar I disorder with a manic episode, (3) no diagnosis of antisocial personality disorder combined with interpersonal violence in the past 2 years, (4) motivated and available for treatment as well as assessments, (5) an IQ above 80, (6) lived within a 45‐min travel distance to the mental healthcare center, (7) a permanent home address, and (8) no Schema therapy (ST) or Dialectical Behavior Therapy (DBT) received in the past year (Wibbelink et al. 2022). Out of the 291 BPD participants who were recruited and screened, 87 participants were not eligible for participation (*n* = 36 unreachable/unmotivated, *n* = 30 BPDSI‐5 score below 20, *n* = 11 no BPD diagnosis, *n* = 7 no BPD as primary diagnosis, *n* = 1 IQ below 80, *n* = 1 psychotic disorder in the past year, and *n* = 1 no permanent home address), resulting in a final sample of 204 participants with BPD.

Participants in the comparison group were included if they met the following criteria: (1) no diagnosis of a personality disorder, (2) no severe psychological complaints in the past 6 months, and (3) no specialized mental healthcare received in the past year. In the comparison group, 86 out of 104 participants who were recruited for the study were included in the present study (*n* = 12 unreachable/unmotivated, *n* = 5 specialized mental health treatment in the past year, and *n* = 1 severe psychological complaints in the past 6 months).

### 2.2 Procedure

The participants with BPD were enrolled in a multicenter RCT on the effectiveness of treatment for BPD (BOOTS study; Wibbelink et al. 2022). The participants were recruited and assessed between January 2019 and October 2021 at various Dutch mental healthcare centers, including Antes (Rotterdam), GGZ inGeest (Amsterdam), GGZ NHN (Heerhugowaard), GGZ Rivierduinen (Leiden), NPI (Amsterdam), Pro Persona (Ede and Tiel), PsyQ (Rotterdam‐Kralingen), and PsyQ/i‐psy (Amsterdam). Individuals diagnosed with BPD or suspected of having BPD were invited to participate in the study. After written informed consent was obtained, the screening process started, involving an assessment with the Dutch versions of the Structured Clinical Interview for DSM‐5 Personality Disorders (SCID‐5‐PD; First et al. 2015) and Structured Clinical Interview for DSM‐5 Clinician Version (SCID‐5‐CV; First et al. 2018). Next, the BPDSI‐5 and a screening interview to evaluate the participant's motivation and availability were administered. Participants eligible for participation were invited for a baseline assessment which included self‐report questionnaires and interviews. Following this, participants were randomized to an evidence‐based treatment for BPD (DBT or ST) and were reassessed over the course of 3 years. In the current study, only the cost interviews and QoL instruments at baseline were included. The BOOTS study was approved by the Medical Ethics Committee. For detailed information on the procedure, the reader is referred to Wibbelink et al. (2022).

Participants in the comparison group were recruited through convenience sampling via online advertisements (e.g., LinkedIn, Facebook) or in person (friends, family, or acquaintances). To minimize differences in costs due to demographic factors, we aimed to recruit participants who matched the BPD sample and a cluster C PD sample (Groot et al. 2022) in terms of gender, age, and education. After recruitment, potential participants were screened for the presence of a personality disorder and utilization of specialized mental healthcare treatment in the past year using two self‐report questions. Additionally, participants were asked about their mental health using one open‐format interview question to screen for severe psychological complaints in the past 6 months. Participants suspected of having a personality disorder or awaiting specialized mental healthcare treatment were deemed noneligible for participation. No formal assessment with validated instruments was conducted. The participants were recruited and assessed between November 2023 and January 2024. The study received ethical approval.

### 2.3 Materials

#### 2.3.1 QoL

QoL was assessed using two measures: EQ‐5D five‐level version (EQ‐5D‐5L; Herdman et al. 2011) and Mental Health Quality of Life seven‐dimensional Questionnaire (MHQoL‐7D; van Krugten et al. 2022). The EQ‐5D‐5L is the most frequently used QoL instrument in studies examining QoL in BPD (IsHak et al. 2013) and assesses five QoL domains, which include mobility, self‐care, usual activities, pain/discomfort, and anxiety/depression. Each domain has five severity levels ranging from 1 = *no problems* to 5 = *extreme problems*/*unable*, and together these scores can classify individuals into 3125 unique health states. To generate health utilities, the health states were weighted by Dutch social tariffs resulting in values ranging from −0.446 (worst imaginable health state) to 1 (best imaginable health state) (Versteegh et al. 2016). Accordingly, the estimated health utility reflects the value that the Dutch population would assign to the participant's health state. Research has demonstrated adequate psychometric properties of the EQ‐5D‐5L among different patient groups and in different countries (Janssen et al. 2013).

The MHQoL‐7D is a recently developed instrument designed to specifically assess QoL in individuals with mental health problems. The MHQoL‐7D measures seven QoL domains: self‐image, independence, mood, relationships, daily activities, physical health, and hope. Each domain is assessed using a 4‐point Likert scale, ranging from 1 = *very satisfied/no problems* to 4 = *very dissatisfied/many problems*. Combining these scores results in 16,348 possible health states. Dutch social tariffs (van Krugten et al. 2024) were used to generate health utilities, ranging from −0.741 (worst imaginable health state) to 1 (best imaginable health state). The MHQoL‐7D has been shown to be a reliable and valid measure in nonclinical and clinical populations and in several countries (Enzing et al. 2022; van Krugten et al. 2022; Wang et al. 2023).

#### 2.3.2 Societal Costs

Societal costs were assessed bottom‐up using a retrospective cost interview especially designed for individuals with BPD (Wetzelaer et al. 2014), with a recall period of 26 weeks for BPD participants and 18 weeks for participants in the comparison group. Cost volumes were multiplied by 2 (BPD group) or 2.89 (comparison group) to estimate annual costs. The cost interview covered healthcare costs, patient and family costs, and costs in other sectors. Costs related to healthcare included visits to psychiatric institutions, private mental healthcare practices, general practice mental health workers, general practitioners, hospitals, social work, paramedical care, complementary therapy, prescribed medication, and others (e.g., rehabilitation care, peer support services, midwife). Patient and family costs comprised informal care (i.e., care provided by the patient's family or friends), out‐of‐pocket costs (e.g., over‐the‐counter medication, alcohol, drugs, impulsive buying), and travel costs. Finally, costs in other sectors included police contacts and productivity losses from unpaid labor (study, volunteer work, and domestic activities) and paid labor. A distinction was made between costs primarily due to psychological complaints (e.g., specialized outpatient treatment in a psychiatric institution, general practice mental health worker, private mental healthcare practice) and costs primarily related to somatic complaints (e.g., physical therapist, midwife, occupational therapist).

To calculate societal costs, Dutch guidelines were followed (Hakkaart‐van Roijen and Kanters 2024). All unit costs were adjusted for the 2024 (June) price level with the consumer price index of the Dutch Central Bureau of Statistics. Healthcare costs were calculated by volume of resource use multiplied by their corresponding unit cost (Hakkaart‐van Roijen and Kanters 2024). In case volumes were missing or unclear, we adopted a conservative approach by estimating the volume as one occurrence. For healthcare services without standardized cost prices, unit costs were based on the Dutch Health Care Authority, professional associations, or a comparable type of healthcare with a standardized cost price. Costs for complementary therapy (e.g., healer, reiki) were used as reported by the participant or, in case reported costs were missing, costs were estimated by applying average prices from the total sample. Prescribed medication costs consisted of medication prices and dispensing costs. Medication prices were calculated using the Daily Defined Dosage (DDD) price from the Dutch Pharmacotherapeutic Compass, excluding VAT, and multiplied by the number of days the medication was used. Additionally, a dispensing fee of 6.96 euros was applied for medication used for 90 days or less, while for medication used for more than 90 days, the dispensing costs were computed proportionally (Hakkaart‐van Roijen and Kanters 2024). In case participants reported only a general medication category (e.g., antibiotics), a pharmaceutical expert was consulted to determine the most common medication within this category (e.g., amoxicillin). Informal care costs were calculated by multiplying the number of hours the participant received informal care by a shadow price. Shadow prices were also used for productivity losses in voluntary work and domestic activities. Productivity losses in education were calculated using standardized prices for the relevant education level or an average price if unavailable, with a maximum of 940 h per year (Hakkaart‐van Roijen and Kanters 2024).

For alcoholic drinks, reference prices from the Dutch Central Bureau of Statistics (StatLine 2019) were used to value wine and beer purchased by the participant or others, distinguishing between purchases made in stores or supermarkets versus bars or restaurants. For distilled beverages, recreational drugs, tobacco, over‐the‐counter medication, and other out‐of‐pocket costs (e.g., impulsive buying, binge eating, costs due to self‐injury), the study relied on the participant‐reported costs. For distilled beverages, recreational drugs, and tobacco for which only quantities were reported without associated costs, costs were estimated by applying average prices from the total sample to the reported volumes. For frequently used over‐the‐counter medication (e.g., paracetamol, ibuprofen), we used standardized prices from a well‐known Dutch drugstore chain. Travel costs were calculated using standardized prices for car usage, including parking fees (Hakkaart‐van Roijen and Kanters 2024). For healthcare services without a standardized travel cost price, travel costs were based on a comparable service in terms of the total number of institutions in the Netherlands. If numbers were unavailable, the travel cost price of physical therapy was used. For police contacts, the hourly wage of police officers, as reported by the Dutch Central Bureau of Statistics (StatLine 2023), was used. Supporting Information S1: Appendix Table A1 presents an overview of the unit costs per cost item.

Finally, costs related to productivity losses in paid labor were calculated using the Friction Cost Approach (FCA). The FCA adopts the employer's perspective and assumes that sick workers can be replaced within a certain time frame (i.e., friction period), therefore, only including costs related to the time required to replace a sick employee (Pike and Grosse 2018). Following FCA, productivity losses due to incapacity to work were calculated only for participants who were employed (i.e., absenteeism), with a friction period of 16.4 weeks and a friction tariff of 42.71 euros (Hakkaart‐van Roijen and Kanters 2024). Absenteeism caused by temporary workplace closures due to COVID‐19 restrictions was left out of the analysis. However, given the ongoing debate about how productivity losses in paid labor should be assessed (Pike and Grosse 2018; van Asselt et al. 2008), we conducted a sensitivity analysis using the Human Capital Approach (HCA). In the HCA, it is assumed that productivity loss due to both illness and disability results in a loss of the individual's productivity to society. Consequently, the potential income individuals could have earned if they had been able to work was included in the cost estimation (Pike and Grosse 2018). We employed two HCA methods in our sensitivity analysis. In the first HCA method (HCA‐1), costs related to absenteeism for employees were computed without a friction period and using the friction tariff. In the second method (HCA‐2), productivity losses were calculated for participants who were employed as well as participants who were unemployed, including participants who received a disability or welfare benefit and students. For unemployed or partly employed participants, we estimated productivity loss using a scenario analysis, assuming that participants without psychological or somatic complaints would pursue a paid job and contribute to societal productivity (Wagner et al. 2022). Accordingly, we multiplied the friction tariff with the average number of hours of an employee (i.e., potential working hours), obtained from the Dutch Central Bureau of Statistics (StatLine 2024) and taking into account gender and age (van Asselt et al. 2007; Wagner et al. 2022). The potential weekly working hours for full‐time students was set at 20.47 (Centraal Bureau voor de Statistiek 2023). In addition, for employed participants, productivity loss encompassed missed hours (absenteeism) as well as the difference between the participant's contractual and potential working hours (Li et al. 2006).

### 2.4 Data‐Analysis

First, descriptive statistics were used to summarize demographics and QoL of both samples. In case QoL data did not follow a normal distribution, bias‐corrected and accelerated (BCa) nonparametric bootstrapping (1000 replications) was also performed to compute BCa 95% confidence intervals (CIs). To compare the two groups, independent‐samples *t* tests were conducted for continuous variables, with BCa bootstrapping (1000 replications) in case of a nonnormal distribution. For categorical variables, *χ* ^2^ tests or Fisher–Freeman–Halton exact tests were used, depending on the expected cell counts. Additionally, effect sizes (Cohen's *d*) for differences in QoL between groups were calculated and interpreted using Cohen's (1992) guidelines (0.20 = small, 0.50 = medium, and ≥ 0.80 = large). Second, although cost data are usually right‐skewed and truncated at zero, arithmetic means combined with nonparametric BCa bootstrapping (1000 replications) are considered appropriate measures to interpret cost data (Barber and Thompson 2000; Ramsey et al. 2005). Subsequently, differences between groups in costs were examined using bootstrapped independent‐samples *t* tests with BCa (Barber and Thompson 2000). Effect sizes (Cohen's *d*) were computed based on bias‐corrected bootstrapped means and standard deviations.

Finally, in case the groups differed in demographics and the demographic was associated with the variable of interest (cost item or QoL) in one or both groups, tested with regression analysis with BCa bootstrapping (1000 replications), between‐group analyses were repeated using a regression model, including group, demographic, and their interaction. The demographic variable was recoded to match the distribution of the BPD sample, ensuring that the effect of group reflected a scenario in which the distribution of the demographic variable in the comparison group was identical to the distribution in the BPD group. A generalized linear regression model with gamma distribution was employed for skewed variables. Employment status was not controlled for, as nonemployment was considered a common contextual factor in individuals with BPD (see Miller and Chapman 2001). The analyses were performed using IBM SPSS (version 28.0.1.0, IBM Corp 2021), with a two‐sided *p*‐value of < 0.05. To take the multiple comparisons into account, the false discovery rate (FDR) correction (Benjamini and Hochberg 1995) was applied to the specific cost items.

## 3 Results

### 3.1 Demographics

Demographic data of the BPD group and comparison group are presented in Table 1, including tests for between‐group differences. Participants with BPD differed significantly from participants in the comparison group with respect to gender and employment status. Therefore, subsequent between‐group analyses were repeated by correcting for gender in case gender was related to the variable of interest in one or both groups.

**Table 1:** Demographic data of the BPD group (*N* = 204) and comparison group (*N* = 86).

Characteristic	BPD		Comparison group		Analysis	
	*M*	*SD*	*M*	*SD*	*t*	*p*
Age	32.21	9.57	34.70	12.67	1.64	0.104
Education^a^	4.19	1.65	4.43	1.56	1.13	0.258
	***N***	**%**	***N***	**%**	**Fisher** b	***p***
Gender					10.48	0.003
Female	172	84.3	58	67.4		
Male	30	14.7	27	31.4		
Other	2	1.0	1	1.2		
Employment status					56.64	< 0.001
Employed	54	26.5	58	67.4		
Employed, sick leave	42	20.6	3	3.5		
Work disabled	42	20.6	3	3.5		
Welfare	19	9.3	3	3.5		
Unemployed	5	2.5	0	0.0		
Student	40	19.6	17	19.8		
Other	2	1.0	2	2.3		

Abbreviation: BPD, borderline personality disorder.

^a^ Based on the International Standard Classification of Education, 2011 version.

^b^ Fisher–Freeman–Halton exact test.

### 3.2 QoL

The average EQ‐5D‐5L index value of the BPD group (*M* = 0.51, *SD* = 0.25, 95% CI = 0.47–0.54) was substantially lower compared with the comparison group (*M* = 0.91, *SD* = 0.12, 95% CI = 0.89–0.93). Similarly, the average MHQoL‐7D index value was considerably lower in the BPD sample (*M* = 0.24, *SD* = 0.35, 95% CI = 0.19–0.29) than in the comparison group (*M* = 0.88, *SD* = 0.14, 95% CI = 0.85–0.91). Since QoL was nonnormally distributed (left skewed) in the comparison group, independent *t* tests with BCa bootstrapping were conducted to examine differences in QoL. The differences in QoL values between the groups were significant and large (EQ‐5D‐5L: *p* < 0.001, *d* = 1.84, MHQoL‐7D: *p* < 0.001, *d* = 2.10). The bias‐corrected descriptive and effect size values from bootstrapping were nearly identical to the values directly derived from the data and therefore not reported. Moreover, as gender was related to QoL, the between‐group analyses were repeated with gamma regression including gender, group, and the interaction between gender and group, which yielded fully consistent results (*p*‐values < 0.001).

### 3.3 Societal Costs

Tables 2, 3, 4 present average annual societal costs for both groups, divided into costs primarily related to psychological complaints, costs primarily related to somatic complaints, and total costs (see Supporting Information S1: Appendix Table A2 for an overview of average annual quantities per cost item). With FCA, bootstrapped average individual total societal costs for BPD participants were €35,038, which was 5.8 times higher than the societal costs of participants without severe psychological complaints (€6081). In the BPD group, 91% (€31,938) of the total societal costs were primarily attributed to psychological problems. In contrast, only 38% (€2320) of the total societal costs in the comparison group were due to psychological complaints. To complement the FCA analysis, a sensitivity analysis was conducted by estimating total societal costs using two different HCA methods (see Table 5). Bootstrapped average individual total societal costs were substantially higher for both groups, varying from €41,783 (HCA‐1) to €68,873 (HCA‐2) in the BPD group and €6278 (HCA‐1) to €20,718 (HCA‐2) in the comparison group.

**Table 2:** Annual individual societal costs (€) in the BPD group (*N* = 204) and comparison group (*N* = 86) due to psychological complaints.

Cost item	BPD			Comparison group			Analysis	
	*M*	Boot *M*	Boot CI	*M*	Boot *M*	Boot CI	*p*	*d* a
*Healthcare costs*	7582.1	7580.0	5916.0–9617.3	166.4	168.3	46.9–339.1	**< 0.001**	0.59
Psychiatric institution^b^	6095.4	6098.3	4590.7–7944.9	138.1	140.5	23.4–310.7	**< 0.001**	0.53
Inpatient care	1490.0	1465.8	659.1–2440.0	0.0	0.0	0.0–0.0	0.056	0.23
Day care	83.1	85.2	2.7–199.2	0.0	0.00	0.0–0.0	0.126	0.15
Outpatient treatment	4348.9	4371.3	3401.9–5606.8	138.1	140.5	23.4–310.7	**< 0.001**	0.62
Home visit	173.5	175.9	52.7–338.6	0.0	0.0	0.0	0.065	0.20
General practice mh worker	26.8	26.7	13.1–42.8	7.9	7.7	0–18.4	0.037	0.21
General practitioner	120.4	119.3	92.2–150.2	6.1	6.0	0–12.2	**< 0.001**	0.54
Hospital	139.0	137.9	55.2–244.1	0.0	0.0	0.0–0.0	0.137	0.21
Inpatient care	87.9	86.9	13.5–189.3	0.0	0.0	0.0–0.0	0.206	0.15
Day care	3.5	3.6	0–7	0.0	0.0	0.0–0.0	0.115	0.11
Emergency care^c^	47.6	47.4	23.3–79.8	0.0	0.0	0.0–0.0	**0.027**	0.27
Social work	696.8	698.4	381.1–1050	0.0	0.0	0.0–0.0	**0.003** d	0.34
Paramedical care^e^	0.8	0.8	0–2.3	0.0	0.0	0.0–0.0	0.112	0.10
Complementary therapy	11.7	11.4	3.7–20.7	0.0	0.0	0.0–0.0	0.084	0.20
Medication	132.7	132.6	99.9–168.7	10.0	9.8	0.6–25.6	**< 0.001**	0.62
Other^f^	358.5	354.7	66.4–731.6	4.3	4.2	0–8.6	0.205	0.17
*Patient and family costs*	11,695.4	11,663.4	9483.4–14,363.0	1588.4	1556.8	928.4–2461.6	**< 0.001**	0.60
Informal care	6017.9	5987.9	3978.6–8406.7	493.8	468.4	25.7–1291.1	**0.009**	0.37
Out‐of‐pocket costs	5404.4	5404.6	4501.5–6576.0	1084.7	1078.5	779.0–1477.0	**< 0.001**	0.66
Otc medication	14.3	14.5	7.2–22.7	1.7	1.7	0.2–3.9	**0.015**	0.26
Substance use	1558.3	1557.6	1317.0–1814.9	864.7	864.3	672.8–1076.2	**< 0.001**	0.45
Other^g^	3831.8	3832.4	2994.8–4937.8	218.3	212.5	33.7–529.6	**< 0.001**	0.59
Travel costs	273.0	270.9	220.5–327.3	9.9	9.9	2.9–19.7	**< 0.001**	0.69
*Costs in other sectors* h	12,725.9	12,737.5	11,213.4–14,322.6	567.4	562.7	163.1–1040.8	**< 0.001**	1.23
Unpaid labor	7476.5	7483.0	6421.7–8644.5	274.6	271.0	97.6–480.8	**< 0.001**	1.02
Volunteer work	118.5	118.7	56.7–197.5	10.5	10.6	0.0–24.2	0.048	0.25
Education	1905.6	1905.7	1181.8–2691.2	73.4	73.5	6.2–183.4	**0.002**	0.39
Domestic activities	5452.4	5449.4	4552.6–6390.1	190.8	188.4	52.1–352.5	**< 0.001**	0.99
Paid labor^h^	5249.4	5270.1	4081.1–6478.8	292.8	285.9	0.0–585.6	**< 0.001**	0.66
*Total costs* h	32,003.4	31,937.8	28,094.4–36,289.3	2322.2	2320.2	1368.9–3485.6	**< 0.001**	1.09

*Note: p* values in bold denote statistical significance at the 0.05 level, with FDR correction applied to the specific cost items.

Abbreviations: Boot CI, bootstrapped 95% confidence interval; Boot *M*, bootstrapped mean; BPD, borderline personality disorder; mh, mental health; otc, over‐the‐counter.

^a^ A positive effect size indicates higher costs in the BPD group versus the comparison group.

^b^ Including specialized psychiatric hospital.

^c^ Including urgent care and ambulance.

^d^ *p* = 0.014 based on the gamma regression analysis including gender, group, and the interaction between gender and group.

^e^ For example, dietitian, occupational therapist, and physical therapist.

^f^ For example, home care, peer support service, and occupational health physician.

^g^ For example, impulsive buying, binge eating, and costs due to self‐injury.

^h^ Costs related to productivity losses in paid labor were determined by the Friction Cost Approach.

**Table 3:** Annual individual societal costs (€) in the BPD group (*N* = 204) and comparison group (*N* = 86) due to somatic complaints.

Cost item	BPD			Comparison group			Analysis	
	*M*	Boot *M*	Boot CI	*M*	Boot *M*	Boot CI	*p*	*d* a
*Healthcare costs*	972.8	978.7	706.1–1276.0	1121.2	1130.4	736.7–1638.2	0.612	−0.07
General practitioner	161.3	160.7	125.3–197.1	80.0	80.0	55.1–107.2	**0.002**	0.34
Hospital	301.5	300.0	202.8–417.6	489.6	499.6	222.4–869.1	0.300	−0.25
Inpatient care	74.4	74.0	13.5–148.8	231.8	240.8	46.4–556.3	0.258	−0.34
Day care	21.1	20.7	3.5–42.2	24.1	24.6	0.0–72.3	0.891^b^	−0.03
Outpatient treatment	124.7	124.3	88.2–161.3	194.3	194.6	112.3–298.0	0.207	−0.28
Emergency care^c^	81.3	81.0	48.5–115.1	39.3	39.6	15.7–62.9	0.081	0.21
Paramedical care^d^	119.0	118.0	66.6–175.9	163.2	166.9	82.4–257.2	0.436	−0.13
Complementary therapy	15.3	15.1	3.6–28.8	16.2	16.5	3.2–31.4	0.937	−0.01
Medication	194.6	196.6	95.6–322.5	257.5	257.1	73.1–511.6	0.665	−0.07
Other^e^	181.1	186.5	34.4–394.8	114.8	110.7	31.0–220.5	0.590	0.06
*Patient and family costs*	183.4	181.6	94.6–296.6	164.7	165.9	90.6–261.1	0.794	0.03
Informal care	106.4	104.5	22.9–219.1	87.3	87.9	26.7–168.4	0.803	0.03
Otc medication	24.5	24.4	13.1–38.3	31.0	31.1	18.3–48.5	0.561	−0.09
Travel costs	52.6	52.8	38.4–72.5	46.5	46.9	31.2–64.8	0.643	0.06
*Costs in other sectors* f	1909.3	1909.9	1343.2–2536.2	2459.4	2467.7	1112.9–4187.3	0.545	−0.14
Unpaid labor	1263.3	1266.5	824.3–1800.5	759.3	765.2	322.4–1407.5	0.221	0.16
Volunteer work	33.3	32.5	9.8–63.3	45.3	45.4	8.2–91.0	0.701	−0.07
Education	212.2	212.8	40.7–462.5	115.5	116.0	25.7–229.7	0.495	0.07
Domestic activities	1017.9	1021.1	628.2–1476.7	598.5	603.9	184.3–1243.2	0.262	0.15
Paid labor^f^	646.0	643.8	364.9–980.1	1700.1	1707.8	739.2–2938.2	0.126	−0.57
*Total costs* f	3065.5	3067.2	2300.4–3910.1	3745.3	3753.3	2196.6–5644.3	0.540	−0.13

*Note: p* values in bold denote statistical significance at the 0.05 level, with FDR correction applied to the specific cost items.

Abbreviations: Boot CI, bootstrapped 95% confidence interval; Boot *M*, bootstrapped mean; BPD, borderline personality disorder; otc, over‐the‐counter.

^a^ A positive effect size indicates higher costs in the BPD group versus the comparison group.

^b^ *p* = 0.645 based on the gamma regression analysis including gender, group, and the interaction between gender and group.

^c^ Including urgent care and ambulance.

^d^ For example, dietitian, occupational therapist, and physical therapist.

^e^ For example, rehabilitation outpatient care, midwife, peer support service, occupational health physician, abortion, and home care.

^f^ Costs related to productivity losses in paid labor were determined by the Friction Cost Approach.

**Table 4:** Total annual individual societal costs (€) in the BPD group (*N* = 204) and comparison group (*N* = 86).

Cost item	BPD			Comparison group			Analysis	
	*M*	Boot *M*	Boot CI	*M*	Boot *M*	Boot CI	*p*	*d* a
*Healthcare costs*	8554.9	8585.2	6707.1–10,700.5	1287.6	1274.0	898.1–1712.3	**< 0.001**	0.58
Psychiatric institution^b^	6095.4	6094.9	4435.4–7990.5	138.1	136.7	24.8–283.3	**0.003**	0.54
Inpatient care	1490.0	1490.0	689.6–2548.1	0.0	0.0	0.0–0.0	0.067	0.24
Day care	83.1	84.0	2.7–179.5	0.0	0.0	0.0–0.0	0.167	0.15
Outpatient treatment	4348.9	4343.3	3391.5–5447.3	138.1	136.7	24.8–283.3	**< 0.001**	0.61
Home visit	173.5	177.6	46.9–334.7	0.0	0.0	0.0–0.0	0.069	0.20
General practice mh worker	26.8	26.6	14.6–41.3	7.9	7.9	1.5–16.9	0.048	0.21
General practitioner	281.7	282.7	231.8–337.5	86.1	86.2	62.6–112.7	**< 0.001**	0.61
Hospital	440.5	444.8	305.9–627.8	489.6	485.0	239.9–785.2	0.795	−0.05
Inpatient care	162.3	166.1	55.7–324.6	231.8	229.6	46.4–509.9	0.649	−0.09
Day care	24.6	25.0	7.0–52.8	24.1	24.2	0.0–72.3	0.979^c^	0.00
Outpatient treatment	124.7	125.1	87.4–166.3	194.3	192.3	110.8–289.4	0.197	−0.30
Emergency care^d^	128.8	128.6	85.2–173.6	39.3	38.9	15.7–62.9	**0.004**	0.34
Social work	696.8	703.2	396.8–1044.8	0.0	0.0	0.0–0.0	**0.007** e	0.34
Paramedical care^f^	119.8	120.8	69.2–181.6	163.2	162.5	92.9–247.2	0.436	−0.12
Complementary therapy	26.9	27.0	9.9–47.0	16.2	16.5	3.2–33.3	0.383	0.10
Medication	327.3	326.2	220.3–456.3	267.5	265.5	91.0–496.1	0.719	0.07
Other^g^	539.6	559.0	196.0–1027.3	119.1	113.6	34.9–225.0	0.133	0.18
*Patient and family costs*	11,878.8	11,836.7	9427.5–14,367.3	1753.1	1716.2	1030.5–2662.3	**< 0.001**	0.61
Informal care	6124.3	6177.2	4018.5–8717.5	581.1	567.6	101.5–1359.8	**0.002**	0.37
Out‐of‐pocket costs	5428.9	5419.6	4432.0–6475.1	1115.7	1114.1	786.9–1525.8	**< 0.001**	0.67
Otc medication	38.8	39.3	22.9–61.9	32.7	32.6	18.9–48.6	0.628	0.06
Substance use	1558.3	1550.4	1335.2–1786.4	864.7	868.0	664.4–1090.7	**< 0.001**	0.45
Other^h^	3831.8	3829.9	2915.0–4838.8	218.3	213.4	31.0–525.2	**< 0.001**	0.59
Travel costs	325.6	326.2	265.9–393.6	56.3	56.1	42.1–73.2	**< 0.001**	0.68
*Costs in other sectors* i	14,668.1	14,680.3	13,002.5–16,501.1	3034.0	3037.7	1645.9–4754.9	**< 0.001**	1.10
Police contacts^j^	32.9	32.9	15.4–55.0	7.3	7.4	1.8–16.4	0.089	0.20
Unpaid labor	8739.8	8755.6	7487.3–10,093.2	1033.9	1019.4	545.8–1611.0	**< 0.001**	0.96
Volunteer work	151.7	152.8	76.7–245.4	55.8	55.8	11.6–115.3	0.093	0.18
Education	2117.8	2118.1	1431.1–2893.4	188.8	187.1	47.7–362.2	**< 0.001**	0.40
Domestic activities	6470.3	6484.7	5505.5–7618.6	789.3	776.6	331.6–1343.1	**< 0.001**	0.86
Paid labor^i^	5895.4	5913.9	4641.7–7193.1	1992.9	1993.6	953.4–3279.7	**< 0.001**	0.50
*Total costs* i	35,101.8	35,038.0	31,272.2–39,141.1	6074.7	6080.8	4365.8–8114.4	**< 0.001**	1.10

*Note: p* values in bold denote statistical significance at the 0.05 level, with FDR correction applied to the specific cost items.

Abbreviations: Boot CI, bootstrapped 95% confidence interval; Boot *M*, bootstrapped mean; BPD, borderline personality disorder; mh, mental health; otc, over‐the‐counter.

^a^ A positive effect size indicates higher costs in the BPD group versus the comparison group.

^b^ Including specialized psychiatric hospital.

^c^ *p* = 0.288 based on the gamma regression analysis including gender, group, and the interaction between gender and group.

^d^ Including urgent care and ambulance.

^e^ *p* = 0.014 based on the gamma regression analysis including gender, group, and the interaction between gender and group.

^f^ For example, dietitian, occupational therapist, and physical therapist.

^g^ For example, rehabilitation outpatient care, midwife, peer support service, occupational health physician, abortion, and home care.

^h^ For example, impulsive buying, binge eating, and costs due to self‐injury.

^i^ Costs related to productivity losses in paid labor were determined by the Friction Cost Approach.

^j^ No distinction was made between costs due to somatic versus psychological complaints.

**Table 5:** Annual individual costs (€) related to productivity losses in paid labor due to psychological or somatic complaints, based on three different approaches.

Method	BPD			Comparison group			Analysis	
	*M*	Boot *M*	Boot CI	*M*	Boot *M*	Boot CI	*p*	*d* a
Psychological								
FCA	5249.4	5270.1	4081.1–6478.8	292.8	285.9	0.0–585.6	**< 0.001**	0.66
HCA‐1	11,797.3	11,779.9	9058.1–14,682.2	292.8	291.3	0.0–585.6	**< 0.001**	0.63
HCA‐2	32,405.6	32,366.4	28,257.0–36,289.6	3712.9	3752.8	1289.2–6791.1	**< 0.001**	1.15
Somatic								
FCA	646.0	643.8	364.9–980.1	1700.1	1707.8	739.2–2938.2	0.126	−0.57
HCA‐1	759.0	755.1	466.6–1121.7	1888.4	1877.3	891.1–3124.9	0.115	−0.49
HCA‐2	2446.5	2467.4	1263.5–3978.4	2531.6	2541.1	1148.1–4169.2	0.926	−0.01
Total								
FCA	5895.4	5913.9	4641.7–7193.1	1992.9	1993.6	953.4–3279.7	**< 0.001**	0.50
HCA‐1	12,556.3	12,535.0	9851.2–15,503.0	2181.1	2168.6	1136.5–3493.2	**< 0.001**	0.57
HCA‐2^b^	39,622.7	39,624.5	36,166.5–42,892.4	16,506.3	16,608.7	12,671.6–20,830.6	**< 0.001**	1.04
Total societal costs								
FCA	35,101.8	35,038.0	31,272.2–39,141.1	6074.7	6080.8	4365.8–8114.4	**< 0.001**	1.10
HCA‐1	41,762.7	41,783.1	36,612.6–47,372.0	6263.0	6278.3	4323.9–8647.4	**< 0.001**	1.08
HCA‐2	68,829.1	68,872.6	63,222.7–74,947.7	20,588.1	20,718.4	16,132.7–25,431.2	**< 0.001**	1.31

*Note:* FCA includes costs related to absenteeism with a friction period of 16.4 weeks. HCA‐1 includes costs related to absenteeism without a friction period, while HCA‐2 includes costs related to absenteeism, unemployment, welfare benefit, disability benefit, and reduced working hours. *p*‐values in bold denote statistical significance at the 0.05 level.

Abbreviations: Boot CI, bootstrapped 95% confidence interval; Boot *M*, bootstrapped mean; BPD, borderline personality disorder; FCA, Friction Cost Approach; HCA, Human Capital Approach.

^a^ A positive effect size indicates higher costs in the BPD group versus the comparison group.

^b^ The total costs exceeded the combined psychological and somatic costs because additional productivity losses for unspecified reasons were also included.

Upon closer examination of both samples, the most significant cost driver of total societal costs was costs in other sectors (BPD group: 42%, comparison group: 35%), specifically productivity losses due to psychological problems (BPD group) or somatic problems (comparison group), followed by patient and family costs (BPD group: 34%, comparison group: 28%), and healthcare costs (BPD group: 25%, comparison group: 21%). Total healthcare costs in BPD participants were primarily driven by treatments in psychiatric institutions or hospitals, particularly outpatient treatment, whereas healthcare costs in the comparison group were mainly attributable to somatic treatments in hospitals. In addition, in the BPD group, total patient and family costs were predominantly driven by informal care related to psychological problems. In the comparison group, out‐of‐pocket costs related to substance use were the main cost driver of total patient and family costs. Furthermore, costs in other sectors were mainly due to productivity losses in unpaid labor (BPD group) or paid labor (comparison group). However, when the HCA methods were employed, productivity loss in paid labor was the most significant cost driver of costs in other sectors in both groups.

Finally, differences between the two groups were examined. The BPD group incurred significantly higher psychological costs for all patient and family cost items and cost items in other sectors, whereas healthcare costs were significantly higher for psychiatric institution, specifically outpatient treatment, consultations with general practitioners, emergency care, social work, and medication. No significant differences were found for somatic costs, except for general practitioner with higher costs in the BPD group compared with the comparison group. In addition, BPD participants demonstrated, in general, significantly higher total costs for patient and family costs and costs in other sectors. For total healthcare costs, significantly higher costs in the BPD group were found for outpatient psychiatric treatment, general practitioner, emergency care, and social work. The between‐group analyses were repeated for cost items that were significantly related to gender using gamma regression, including gender, group, and the interaction between gender and group, which yielded comparable results.

## 4 Discussion

The present study investigated the burden of disease of BPD among Dutch treatment‐seeking outpatients, focusing on QoL and societal costs. A comprehensive evaluation was performed by estimating annual costs from a societal perspective using a bottom‐up approach and differentiating between costs primarily related to psychological versus somatic problems. Moreover, QoL was determined using a well‐known generic QoL measure (EQ‐5D‐5L) as well as a measure specifically designed for individuals with mental health problems (MHQoL‐7D). Additionally, the findings were compared with a comparison group without severe psychological complaints. Our results indicate a severely impaired QoL combined with substantial societal costs for BPD outpatients, which were markedly different from the comparison group.

Using the generic QoL measure (EQ‐5D‐5L), BPD outpatients reported a severely impaired QoL (0.51), which was substantially lower compared with the comparison group (0.91). Moreover, QoL in our BPD sample was comparable with findings from previous studies among individuals with BPD (Bales et al. 2012; Laurenssen et al. 2016; McMain et al. 2012; Soeteman et al. 2008; van Asselt et al. 2008), and lower compared with the utility scores of the general Dutch population (0.87; Versteegh et al. 2016) and individuals with other mental disorders (e.g., depressive disorder; Woo et al. 2014, anxiety disorder; Franklin and Hernández Alava 2023) or somatic disorders (e.g., obesity, chronic kidney disease, cardiovascular disease, diabetes; Zhou et al. 2021). To complement the generic EQ‐5D‐5L, a QoL measure tailored to individuals with mental health problems (MHQoL‐7D) was employed in the current study, revealing an even greater impairment in QoL among BPD outpatients (0.24). Although population norms are not available, QoL was substantially lower compared with the comparison group (0.88) and slightly lower compared with individuals with cluster C PD (0.35; Groot et al. 2025). The greater impairment in QoL observed with the MHQoL‐7D compared with the EQ‐5D‐5L, along with the larger difference with the comparison group, suggests that the MHQoL‐7D is a more sensitive measure for evaluating QoL among individuals with (severe) psychological problems. It is therefore recommended to use the MHQoL‐7D alongside generic QoL measures for a comprehensive assessment of QoL in individuals with mental disorders (Enzing et al. 2022).

The estimated societal costs associated with BPD were €35,038 per individual per year, primarily driven by costs in other sectors (€14,680; 42%), followed by patient and family costs (€11,837; 34%) and healthcare costs (€8585; 25%). Moreover, societal costs were largely attributable to psychological problems (91%). Societal costs were determined by applying the FCA to productivity losses in paid work, in accordance with guidelines of Hakkaart‐van Roijen and Kanters (2024). However, the FCA has been criticized by several authors as it includes productivity costs related to absenteeism within a defined period (friction period), thereby capturing only actual production losses (Pike and Grosse 2018). Productivity costs associated with work disability are by definition zero in the FCA, because the friction period has already passed. Given that disabled individuals represent a cost to society, the FCA might not fully capture true societal costs (van Asselt et al. 2008). With more than half of the BPD participants not employed in our sample, the FCA likely underestimated the societal costs of BPD. Therefore, we also employed two HCA methods to estimate societal costs. The first method (HCA‐1) included absenteeism without a maximum period, while the second method (HCA‐2) included absenteeism, productivity losses due to disability or unemployment, and reduced working hours (Li et al. 2006). The societal costs based on the HCA were markedly higher for both groups, ranging from €41,783 (HCA‐1) to €68,873 (HCA‐2) in the BPD group and €6278 (HCA‐1) to €20,718 (HCA‐2) in the comparison group. Regardless of whether the FCA or HCA was used, the high estimated societal costs of BPD outpatients underscore the substantial economic burden of BPD.

The economic burden of BPD in our sample (FCA: €35,038; HCA‐1: €41,783; HCA‐2: €68,873) was somewhat higher compared with other studies on BPD (Hastrup et al. 2019; Laurenssen et al. 2016; Salvador‐Carulla et al. 2014; van Asselt et al. 2007; Wagner et al. 2014, 2022). In studies using the HCA, societal costs of BPD ranged from €17,704 (Salvador‐Carulla et al. 2014) to €52,452 (Hastrup et al. 2019), whereas the FCA‐based study by Laurenssen et al. (2016) reported societal costs of €22,095^1^. The study by van Asselt et al. (2007) was also conducted among Dutch outpatients and employed a methodology largely similar to our study. Consistent with our study, nonhealthcare costs formed a large part of the total societal costs (78%). However, the estimated societal costs in van Asselt et al. (2007; €29,727) were considerably lower compared with our study. The comparatively higher societal costs in our study may be attributed to the inclusion of costs primarily related to somatic problems, whereas most studies only included costs related to the patient's psychological problems. As somatic costs in individuals with BPD could be a direct or indirect consequence of the disorder (Castle 2019), neglecting costs related to somatic problems can lead to an underestimation of BPD‐related costs, thereby resulting in an incorrect estimation of the overall economic burden of individuals with BPD (Wagner et al. 2022).

The societal costs associated with BPD were considerably higher compared with other mental and somatic disorders (e.g., addiction, anxiety disorders, mood disorders, epilepsy, Parkinson's disease, traumatic brain injury; Gustavsson et al. 2011). Moreover, healthcare costs for BPD outpatients were substantially higher than the average healthcare costs of Dutch individuals aged 20–45 with a mental disorder (€8585 vs. €1709; Rijksinstituut voor Volksgezondheid en Milieu 2019). The greater costs of BPD might be explained by the chronic and complex nature of BPD, including impulsive behavior, fear of abandonment, nonsuicidal self‐injury, suicidal behavior, and high rates of comorbid mental disorders. In addition, societal costs of BPD outpatients were almost six times higher compared with the comparison group without severe psychological problems. The BPD sample demonstrated, in general, higher patient and family costs and costs in other sectors, while healthcare costs were only elevated for outpatient psychiatric treatment, consultations with general practitioners, emergency care, and social work. The increased societal costs of the BPD sample were mainly attributable to their psychological problems, as costs related to somatic complaints were not elevated in the BPD sample compared with the comparison group, except for consultations with the general practitioner. This finding contradicts the results of Hastrup et al. (2019), who reported higher somatic healthcare costs in BPD patients compared with a comparison group. Moreover, Hastrup et al. (2019) found that the societal costs of BPD patients were 16 times higher compared with controls. The differences between our findings and those of Hastrup et al. (2019) might be related to our comparison group. Severe somatic problems were present in our comparison sample, with a prevalence rate higher compared with the general Dutch population. For instance, two participants in the comparison group (2.3%) underwent gastric reduction surgery, whereas only 0.08% of the Dutch population undergo this surgery annually (Poelemeijer et al. 2020). This might have resulted in relatively high societal costs in the comparison group, as evidenced by the higher societal costs in our comparison group (€6081) compared with the comparison group of Hastrup et al. (2019; €3165).

This study has several limitations that need to be acknowledged. A first limitation relates to our measurement of costs, which relied on retrospective questioning and is therefore susceptible to recall bias (Bowling 2014). Different recall periods were used for BPD outpatients (26 weeks) compared with the comparison group (18 weeks), which may have resulted in differences in the precision of responses between the groups and, consequently, could have affected our comparisons. Moreover, information on resource consumption was not based on objective sources such as data from healthcare providers and insurance companies. In the study by Hall et al. (2001), inconsistencies were found between the number of hospital admissions recorded by hospitals and those reported by patients with BPD. However, individuals with BPD utilize a wide variety of mental healthcare providers, making it infeasible to collect data from objective sources (van Asselt et al. 2007). Moreover, several costs particularly relevant to BPD (e.g., out‐of‐pocket costs, informal care) cannot be collected through objective sources. Second, we estimated prescribed medication costs using the DDD, consistent with other cost‐of‐illness studies (e.g., Bodden et al. 2022; Groot et al. 2022; Sveen et al. 2023). The DDD is, however, a rough estimate of medication consumption and there may have been discrepancies between the actual daily dose of participants and the DDD, potentially leading to misestimation of medication costs. Nevertheless, medication prices were relatively low, particularly when compared with the dispensing costs.

Third, BPD participants were recruited and assessed between 2019 and 2021, while participants in the comparison group were recruited and assessed between 2023 and 2024, potentially introducing history effects. Moreover, data from several BPD participants were collected during the COVID‐19 pandemic, which may have influenced the cost estimates for the BPD outpatients and, as a result, affected their comparability with the comparison group. For example, nonessential healthcare was postponed and restaurants were closed for several months during the COVID‐19 pandemic, which might have led to an underestimation of costs. In contrast, costs related to productivity loss in paid work might be overestimated, as employees were not permitted to work at their workplace with a minor cold, potentially resulting in higher absenteeism. Additionally, some participants might have worked fewer hours due to the temporary closing of their workplaces (e.g., restaurant, museum, hair salon). However, if participants indicated that their reduced hours were due to COVID‐19 restrictions, those hours were left out of the analysis.

Fourth, cost estimates of individuals with BPD were based on outpatients seeking specialized treatment in the context of an RCT (the BOOTS study; Wibbelink et al. 2022). Including treatment‐seeking patients may have led to an overestimation of costs, as they might have experienced a higher burden of distress while waiting for psychotherapy. On the other hand, costs could also have been underestimated, as individuals who do not seek outpatient treatment may incur higher costs in other areas, such as inpatient mental healthcare (Wagner et al. 2014, 2022). Moreover, the context of an RCT may have influenced the cost estimates, as the inclusion and exclusion criteria applied could have introduced selection bias. Individuals with BPD were only included in the BOOTS study if they scored above a certain threshold on a BPD severity measure, indicating the presence of considerable symptoms. However, other individuals with BPD were excluded from the study, such as patients from other settings (e.g., inpatient care, forensic care), as well as patients with psychotic disorders or those requiring clinical detoxification. Additionally, while the BOOTS study was quite inclusive in terms of comorbid disorders—reflecting clinical practice—this also makes it challenging to disentangle the burden specifically attributable to BPD from the effect of other disorders. For example, depression and anxiety disorders, both highly prevalent in BPD (Tomko et al. 2014), are known to contribute considerably to the burden of disease (GBD 2019 Mental Disorders Collaborators 2022). However, as comorbidity is inherent to BPD (Shen et al. 2017; Tomko et al. 2014), including comorbid conditions provides a more accurate representation of the burden of disease of BPD, thereby enhancing the ecological validity of the findings.

Fifth, the participants in the comparison group were selected using convenience sampling, which means that they may not be representative of the population (Bryman 2016). Moreover, in the comparison group, the absence of a personality disorder and severe psychological complaints was determined on self‐report rather than formal assessment, potentially affecting the validity of the comparison group. Furthermore, differences in psychiatric institutional costs may be partly attributable to the comparison group's exclusion criteria, which excluded participants who had received specialized mental healthcare. Lastly, our findings are specific to the Dutch healthcare and social welfare context, limiting the generalization to other countries. For example, inpatient mental healthcare is less common in the Netherlands than in Germany, with Dutch patients experiencing shorter inpatient stays on average. Consequently, studies among Dutch BPD patients reported substantially lower costs for inpatient mental healthcare compared with studies among German BPD patients (Wagner et al. 2014, 2022).

In conclusion, this study demonstrated that BPD is related to severely impaired QoL and substantial societal costs. Societal costs were mainly attributable to costs in other sectors and patient and family costs, indicating that the impact of BPD extends far beyond the healthcare sector. The high economic burden of BPD relative to other mental and somatic disorders, along with low QoL, underscores the detrimental effect of limited treatment access on both individuals with BPD and society. As healthcare policymakers consider the burden of disease of patient populations when prioritizing treatment reimbursement (Stolk et al. 2005), our findings advocate for an increased prioritization of, and consequently greater availability of, specialized treatments for BPD. Our data suggest that this priority would concurrently improve the well‐being of individuals with BPD and reduce societal costs.

## Ethics Statement

The BOOTS study was approved by the Medical Ethics Committee of the Academic Medical Center Amsterdam (registration number NL66731.018.18). The study including the comparison group received approval from the Ethics Review Board of the Faculty of Social and Behavioural Sciences, University of Amsterdam (registration number FMG‐5576_2023).

## Consent

Signed informed consents were obtained from all participants in the study.

## Conflicts of Interest

The authors declare no conflicts of interest.

## Supporting information

Supporting information.

## Data Availability

The participant‐level dataset may contain information that compromises the anonymity of the participants; therefore, the dataset will not be made publicly available. The data supporting the findings of this study can be obtained from the corresponding author (CJMW) upon reasonable request.

## Endnotes

1 To enable comparisons between studies, all reported societal costs were adjusted for the 2024 (June) price level with the consumer price index.
