# Supplementary material for: Burden of Disease of Borderline Personality Disorder: A Comprehensive Evaluation of Quality of Life and Societal Cost of Illness
Source: J Clin Psychol. 2025 Jul 10;81(9):832–46. doi: 10.1002/jclp.70000 (PMC12401484; doi:10.1002/jclp.70000)
Supplement: Supplementary file 1 — Supporting information. [file JCLP-81-832-s001.docx]

**Appendix A**

Table A1

*Overview of Unit Costs per Cost Item*

| Cost item | Unit | Unit cost (€) |
| --- | --- | --- |
| *Healthcare costs* |  |  |
| Psychiatric institution |  |  |
| Inpatient care^1^ | Day | 350 |
| Day care | Day | 182 |
| Specialized outpatient treatment^2^ | Visit | 144 |
| Basic outpatient treatment^3^ | Visit | 130 |
| Outpatient crisis service | Minute | 2 |
| Home visit | Visit | 181 |
| Specialized psychiatric hospital |  |  |
| Inpatient care | Day | 525 |
| Day care | Day | 273 |
| Private mental healthcare practice, psychiatrist | Visit | 150 |
| Private mental healthcare practice, psychologist | Visit | 106 |
| General practice mental health worker | Visit | 22 |
| General practitioner, psychological/somatic complaint | Consultation | 45/33 |
| General practitioner, psychological/somatic complaint | Home visit | 52/46 |
| Hospital |  |  |
| Inpatient care | Day | 690 |
| Day care | Day | 359 |
| Outpatient treatment | Visit | 129 |
| Urgent care | Visit | 192 |
| Emergency care | Visit | 276 |
| Ambulance | Incident | 565 |
| Social work | Visit | 136 |
| Dietitian | Visit | 26 |
| Occupational therapist | Visit | 26 |
| Physical therapist | Visit | 42 |
| Chiropractor | Visit | 60 |
| Remedial therapist | Visit | 46 |
| Movement disorder specialist (“atrokinesioloog”) | Visit | 90 |
| Paramedical care, unspecified | Visit | 39 |
| Haptonomist | Visit | 95 |
| Occupational health physician | Visit | 129 |
| Work integration | Visit | 129 |
| Rehabilitation outpatient care | Visit | 532 |
| Midwife | Visit | 24 |
| Abortion | Visit | 478 |
| Abortion consult | Visit | 49 |
| Care farm | Half-day | 57 |
| Home care | Hour | Various^4^ |
| Sexually Transmitted Diseases test | Incident | 141 |
| Peer support service^5^ | Various | Various |
| Medication | DDD price with fee | Various |
| Complementary therapy | Visit | As reported |
|  |  |  |
| *Patient and family costs* |  |  |
| Informal care |  |  |
| Out-of-pocket costs^6^ |  |  |
| Beer, store/bar | Glass | 0.46/3 |
| Wine, store/bar | Glass | 0.55/5 |
| Travel costs |  |  |
| General practitioner | Visit | 5 |
| Pharmacy | Visit | 5 |
| Physical therapist | Visit | 5 |
| Midwife | Visit | 6 |
| Nursing home | Visit | 6 |
| Hospital | Visit | 7 |
|  |  |  |
| *Costs in other sectors* |  |  |
| Police contacts | Incident | 54 |
| Productivity losses unpaid labor |  |  |
| Volunteer work | Hour | 20 |
| Education | Hour | Various^7^ |
| Domestic activities | Hour | 20 |

*Note.* Cost unit prices were rounded to whole numbers, if possible, to enhance readability. DDD = Daily Defined Dosage.

^1^Also applied to addiction care and crisis service.

^2^Also applied to addiction care.

^3^Also applied to family support.

^4^Cleaner (€35), domestic helper (€68), and home care assistant (€101).

^5^Peer support services included peer support groups and psychological support hotlines. Unit costs were based on shadow prices, adjusted for number of participants and duration.

^6^Costs of other types of out-of-pocket costs, including distilled beverages, recreational drugs, tobacco, over-the-counter medication, and other (e.g., impulsive buying, binge eating) were used as reported.

^7^Secondary vocational education (€22), higher professional education (€18), university education (€34), and other or unknown (€25).

Table A2

*Overview of Annual Individual Quantities per Cost Item in the BPD group (N = 204) and Comparison Group (N* = *86)*

| Cost item | Unit of Quantity (*M*) |  | Quantity (*M*) | | | | | | | | |  |
| --- | --- | --- | --- | --- | --- | --- | --- | --- | --- | --- | --- | --- |
|  |  |  | BPD | | | |  | | Comparison group | | | |
|  |  |  | Psychological | Somatic | Total |  | | Psychological | | Somatic | Total |  |
| *Healthcare costs* |  |  |  |  |  |  | |  | |  |  |  |
| Psychiatric institution^1^ |  |  |  |  |  |  | |  | |  |  |  |
| Inpatient care | Days |  | 4.1 | NA | 4.3 |  | | 0.0 | | NA | 0.0 |  |
| Day care | Days |  | 0.4 | NA | 0.4 |  | | 0.0 | | NA | 0.0 |  |
| Outpatient treatment | Visits |  | 30.0 | NA | 30.0 |  | | 1.1 | | NA | 1.1 |  |
| Home visit | Visits |  | 1.0 | NA | 1.0 |  | | 0.0 | | NA | 0.0 |  |
| General practice mh worker | Visits |  | 1.2 | NA | 1.2 |  | | 0.4 | | NA | 0.4 |  |
| General practitioner | Contacts |  | 2.6 | 4.9 | 7.5 |  | | 0.1 | | 2.4 | 2.5 |  |
| Hospital |  |  |  |  |  |  | |  | |  |  |  |
| Inpatient care | Days |  | 0.1 | 0.1 | 0.2 |  | | 0.0 | | 0.3 | 0.3 |  |
| Day care | Days |  | 0.01 | 0.06 | 0.07 |  | | 0.0 | | 0.1 | 0.1 |  |
| Outpatient treatment | Visits |  | 0.0 | 1.0 | 1.0 |  | | 0.0 | | 1.5 | 1.5 |  |
| Urgent or emergency care | Visits |  | 0.2 | 0.3 | 0.5 |  | | 0.0 | | 0.2 | 0.2 |  |
| Ambulance | Transports |  | 0.01 | 0.01 | 0.02 |  | | 0.0 | | 0.0 | 0.0 |  |
| Social work | Visits |  | 5.1 | NA | 5.1 |  | | 0.0 | | NA | 0.0 |  |
| Paramedical care | Visits |  | 0.03 | 2.8 | 2.8 |  | | 0.0 | | 3.4 | 3.4 |  |
| Complementary therapy | Visits |  | 0.1 | 0.2 | 0.3 |  | | 0.0 | | 0.2 | 0.2 |  |
| Medication | Number |  | 1.1 | 0.8 | 1.9 |  | | 0.1 | | 0.9 | 0.9 |  |
| Other^2^ | Various^3^ |  | 6.5 | 2.8 | 9.3 |  | | 0.2 | | 1.2 | 1.4 |  |
|  |  |  |  |  |  |  | |  | |  |  |  |

| *Patient and family costs*^4^ |  |  |  |  |  |  |  |  |  |
| --- | --- | --- | --- | --- | --- | --- | --- | --- | --- |
| Informal care | Hours |  | 299.0 | 5.3 | 304.2 |  | 24.5 | 4.3 | 28.9 |
| Travel costs | Visits |  | 40.1 | 9.6 | 49.7 |  | 1.6 | 7.9 | 9.5 |
|  |  |  |  |  |  |  |  |  |  |
| *Costs in other sectors*^5^ |  |  |  |  |  |  |  |  |  |
| Police contacts^6^ | Incidents |  | NA | NA | 0.6 |  | NA | NA | 0.1 |
| Unpaid labor | Hours |  | 349.4 | 60.5 | 410.0 |  | 10.7 | 37.6 | 48.2 |
| Volunteer work | Hours |  | 5.8 | 1.6 | 7.5 |  | 0.5 | 2.3 | 2.8 |
| Education | Hours |  | 72.7 | 8.3 | 81.0 |  | 0.7 | 5.6 | 6.2 |
| Domestic activities | Hours |  | 270.9 | 50.6 | 321.4 |  | 9.5 | 29.7 | 39.2 |
| Paid labor^5^ | Hours |  | 122.9 | 15.1 | 138.0 |  | 6.9 | 39.8 | 46.7 |

*Note.* BPD = borderline personality disorder; NA = not applicable.

^1^Including specialized psychiatric hospital.

^2^E.g., rehabilitation outpatient care, midwife, home care, peer support service, occupational health physician, abortion, and home care.

^3^Visits (haptonomist, occupational health physician, work integration, rehabilitation outpatient care, midwife, abortion consult, abortion), half-days (care farm), hours (home care), incidents (Sexually Transmitted Diseases test), or contacts (peer support service).

^4^Other cost items within this category consisted of reported costs.

^5^Productivity losses in paid labor were determined by the Friction Cost Approach.

^6^No distinction was made between costs due to somatic versus psychological complaints.
